# Contrasting the impact of cytotoxic and cytostatic drug therapies on tumour progression

**DOI:** 10.1371/journal.pcbi.1007493

**Published:** 2019-11-18

**Authors:** Jani V. Anttila, Mikhail Shubin, Johannes Cairns, Florian Borse, Qingli Guo, Tommi Mononen, Ignacio Vázquez-García, Otto Pulkkinen, Ville Mustonen

**Affiliations:** 1 Organismal and Evolutionary Biology Research Programme, University of Helsinki, Helsinki, Finland; 2 Department of Computer Science, University of Helsinki, Helsinki, Finland; 3 Department of Epidemiology and Biostatistics, Memorial Sloan Kettering Cancer Center, New York, New York, United States of America; 4 Department of Statistics, Columbia University, New York, New York, United States of America; 5 Helsinki Institute for Information Technology, University of Helsinki, Helsinki, Finland; 6 Institute of Biotechnology, University of Helsinki, Helsinki, Finland; University of Minnesota, UNITED STATES

## Abstract

A tumour grows when the total division (birth) rate of its cells exceeds their total mortality (death) rate. The capability for uncontrolled growth within the host tissue is acquired via the accumulation of driver mutations which enable the tumour to progress through various hallmarks of cancer. We present a mathematical model of the penultimate stage in such a progression. We assume the tumour has reached the limit of its present growth potential due to cell competition that either results in total birth rate reduction or death rate increase. The tumour can then progress to the final stage by either seeding a metastasis or acquiring a driver mutation. We influence the ensuing evolutionary dynamics by cytotoxic (increasing death rate) or cytostatic (decreasing birth rate) therapy while keeping the effect of the therapy on net growth reduction constant. Comparing the treatments head to head we derive conditions for choosing optimal therapy. We quantify how the choice and the related gain of optimal therapy depends on driver mutation, metastasis, intrinsic cell birth and death rates, and the details of cell competition. We show that detailed understanding of the cell population dynamics could be exploited in choosing the right mode of treatment with substantial therapy gains.

## Author summary

Cells and organisms evolve to better survive in their environments and to adapt to new challenges. Such dynamics manifest in a particularly problematic way with the evolution of drug resistance, which is increasingly recognized as a key challenge for global health. Thus, developing therapy paradigms that factor in evolutionary dynamics is an important goal. Using a minimal mathematical model of a cancer cell population we contrast cytotoxic (increasing death rate) and cytostatic (decreasing birth rate) treatments while keeping the effect of the therapy on the net growth reduction constant. We then quantify how the choice and the related gain of optimal therapy depends on driver mutation, metastasis, intrinsic cell birth and death rates and the details of cell competition. Most importantly, we identify specific cell population dynamics under which a certain treatment could be significantly better than the alternative.

## Introduction

Cancer progression is an evolutionary process where cell lineages (clones) acquire somatic mutations due to exogenous (e.g. UV light) and endogenous (e.g. DNA repair deficiency) causes [1]. Cancer driver mutations endow a competitive advantage to a cell, which leads to the corresponding lineage gaining in frequency within the population. The numbers of rate-limiting driver mutations required for tumour development were originally predicted using epidemiological age-incidence curves [2] and subsequently confirmed based on protein and DNA sequence data [3, 4]. For instance, tumours have an estimated four driver substitutions, with some tumour type specific variability [4]. Allowing for additional events from copy number and epigenetic drivers, these numbers are consistent with the hallmarks of cancer comprising six biological capabilities acquired during the multistep progression of tumours [5]. The main hallmarks are sustaining proliferative signaling, evading growth suppressors, resisting cell death, enabling replicative immortality, inducing angiogenesis, and activating invasion and metastasis.

Although the big picture of tumour progression is effectively conceptualized by the hallmarks, important questions about the dynamics are not known and likely depend on cancer type as well as developmental stage. A temporal view of progression across cancers can be sought using large cohorts of genomic data [6]. However, genomic data alone offers no direct measurement of intrinsic birth and death rates, and important ecological variables such as absolute population sizes or modes of competition within the cell population. As it stands, there is no consensus on the details of progression dynamics of tumours through the various stages (see e.g. [7] and its critique). Resolving tumour growth characteristics quantitatively requires more ecological (phenotypic) data to be collected from growing tumours together with measurements of birth and death rates of tumour cells at various stages.

Using drugs to treat cancer has a long history coupled with current rapid development. Classically, effective drug treatment has relied on large enough doses of a cytotoxic agent that kills rapidly dividing cells, resulting in clear decline of tumour. This is not always attainable, however, as most such agents are not cancer cell specific, and often cause severe side-effects. More recently, targeted drugs with cancer cell specificity have been introduced to clinical practice. Most of these operate primarily as cytostatics, disrupting cell signaling and replication, but do not lead to an immediate decrease of tumour burden [8]. The distinction between cytotoxic and cytostatic drugs is not clear cut in actual medication: cytotoxic compounds can also induce stasis with low doses and on apoptosis-resistant cells, and cytostatic effects often also result in cell death on cells in any other than the quiescent phase [9]. However, these two modes of action have different effects on the underlying cell population dynamics.

Many properties of tumour growth and cancer progression are dependent not only on the overall growth rate (difference between births and deaths) of cancer cell population, but more specifically on the birth rate and death rate (hereby denoted as *β*_0_ and *δ*_0_, respectively) of cells. Higher rates give rise to faster cell turnover, and their ratio *q* = *δ*_0_/*β*_0_ yields the probability that a population starting from a single cell faces stochastic extinction [10]. Recent research has linked turnover to spatial heterogeneity [11] and mutational potential [12] within a tumour. This suggests that a cytostatic effect reducing cell reproduction, and a cytotoxic effect increasing cell death may have differential impact on cancer progression, even when their effect on overall cell growth rate is the same.

**Fig 1:**
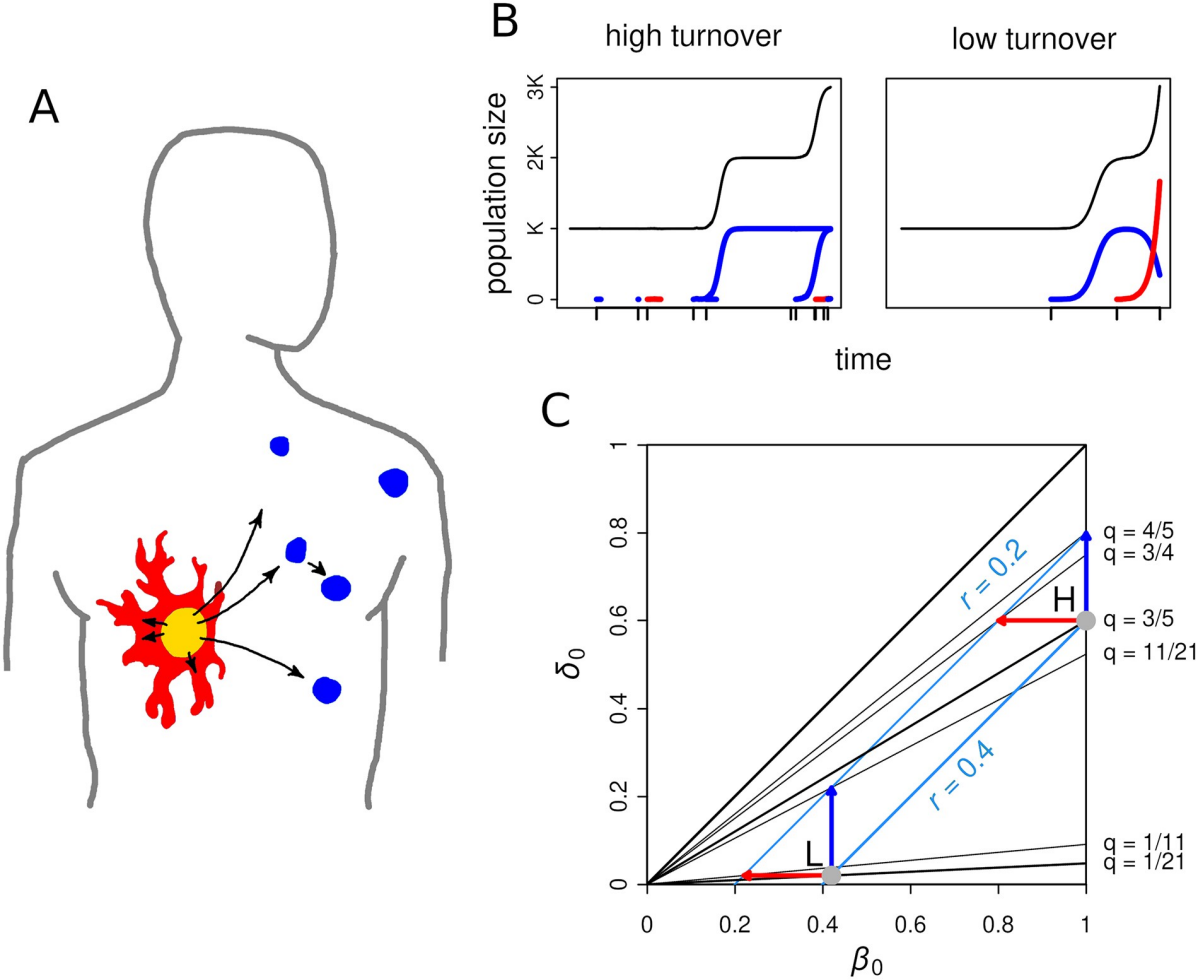
Modelling cancer progression via metastasis and/or driver mutation events. **A** Primary tumour is assumed to have reached its present growth potential (yellow). It can progress to the next epoch of growth via *de novo* driver mutation that, for example, induces angiogenesis (red), or via seeding metastasis (blue). **B** Simulated example trajectories at high (low) cell turnover with *β*_0_ = 1.0, *δ*_0_ = 0.6, (*β*_0_ = 0.42; *δ*_0_ = 0.02). Black line shows total population size. Blue line shows metastatic tumour population sizes and red line shows cell population containing driver mutation. Tick marks on the time axis show metastasis and mutation events, some of which go extinct due to fluctuations that are stronger in high turnover tumours. **C** The simulation experiment setting on a birth rate–death rate plane. The light grey dots marked H and L show the high turnover and low turnover cases, respectively. The arrows show the changes after applying cytostatic (red) or cytotoxic (blue) medication with magnitude Δ = 0.2, which keeps the overall growth reduction constant. The net growth rate *r* (light blue lines) and stochastic extinction risk *q* = *δ*_0_/*β*_0_ (thin black lines) of each scenario is also shown.

Here, we construct a model of tumour progression to quantify and understand such a differential impact so that it could be exploited in therapy choice. We explicitly factor in different modes of competition and intrinsic evolutionary regimes within a cell population to acknowledge the uncertainties we currently have about real tumours. In particular, we focus on the penultimate stage of tumour progression where the malignant cell population is physically detectable comprising ∼ 10^6^ to 10^8^ cells corresponding to ∼1 mm to 6 mm diameter tumour [13]. By this stage, clinical symptoms generally appear, and a tumour reaches its present growth potential, but has yet to enter the final (fatal) epoch of growth. We consider two alternative modes to enter the last epoch of growth (see Fig 1). A driver mutation which, e.g., induces angiogenesis for the primary tumour thus increasing its overall growth potential, can be discovered. Alternatively, the cells in the primary tumour can migrate and seed a metastasis that similarly leads to an overall growth potential increase. We note that the seeding of metastasis is a complex, incompletely understood, process that can either happen linearly, after the primary tumour has become malignant, or in parallel with the progression of the primary tumour. Evidence for both modes has been found across tumour types [14], and our setting is consistent with the parallel model. Purely mathematically, we study how best to influence two stochastic processes where one couples to cell births and the other to cell number under different population dynamics defined by competition and turnover.

Mathematical study of drug therapies in cancer and beyond is thriving. Considerable progress has been made at several fronts including drug combinations [15–19], alternative dosing schedules [20], modifying the objective of therapy from eradication to control when the former is not attainable [21–23], and highlighting the value of frequent monitoring to guide treatment [24]. For cancer therapy, some models have already played a role in clinical success, e.g., optimising imatinib dosing schedules to avert resistance in the treatment of chronic myeloid leukaemia [25], and an application of adaptive therapy to castrate-resistant prostate cancer patients [26]. Here we show that choosing a specific mode of action, cytostatic or cytotoxic, leads to a substantial efficacy gain. We quantify how this choice and the related efficacy gain depends on driver mutation, metastasis, intrinsic cell birth and death rates, and the details of cell competition.

## Results

### Mathematical model of tumour progression

The model system consists of cell populations *n*_*i*,*j*_(*t*) in patches *i* and having phenotypes *j*. The system evolves through four kinds of reactions: *births*, *deaths*, *migrations*, and *mutations* (Table 1). The simulations are initiated with a single (i.e. primary) tumour with a resident cell population *n*_1,1_ at a carrying capacity *K*. From this initial state the total population size can increase substantially by either a) successful migrations to new patches, resulting in metastases, or b) finding a (driver) mutation that entails a higher carrying capacity (Fig 1). These events arise stochastically depending on migration rate *μ* and mutation probability *γ* at cell reproduction.

**Table 1:** Model system reactions. The model system evolves through four possible reactions, according to their *per cell* propensities. C_*i*,*j*_ denotes an individual cell of phenotype *j* in patch *i*. Birth rate *β* and death rate *δ* are functions of total cell population *n*_*i*,·_ in a patch.

reaction		propensity
birth	C_*i*,*j*_ → C_*i*,*j*_ + C_*i*,*j*_	(1 − *γ*)*β* (*n*_*i*,*j*_)
death	C_*i*,*j*_ → ∅	*δ*(*n*_*i*,*j*_)
migration	C_*i*,*j*_ → C_*k*,*j*_	*μ*
mutation	C_*i*,*j*_ → C_*i*,*j*_ + C_*i*,*l*_	*γ* *β*(*n*_*i*,*j*_)

In a migration event, a single cell is moved from a pre-existing patch into a new empty, previously uncolonised patch, of which there is an unlimited supply. This cell then initiates a new metastasis, which may grow in population size, and is subject to stochastic extinction risk. Migrations occur at a rate *μ* per cell.

The mutation events are tied to birth events such that at each birth there is a probability *γ* that the daughter cell is of a different phenotype than its parent. Here, we consider only a single kind of driver mutation, with the sole effect that the mutated population has a higher carrying capacity *L* > *K*. Although mutations affecting various properties occur in reality, major steps in tumour progression are reached by driver mutations that overcome the previous limits to growth. These are most simply represented in our model by changing the carrying capacity parameter while keeping all else equal.

Note that the products *μ K* and *β γ K* are important, since these yield the metastasis and mutation rates, whereas the actual value of *K* is of less importance (provided it is large compared to the stochastic extinction barrier at ∼ few tens of cells, a condition which is easily fulfilled for tumours). In our simulations, the mutated subpopulations face competitive pressure from the resident population. However, the value of *L* does not matter for our analysis in so far it is much bigger that *K*, which we assume to be the case for an epoch changing event. Ecologically interpreted this would mean that these cells can enter a new niche or exploit a new resource.

Cell population growth is assumed to follow a modified version of logistic growth, following [27], which allows for adjusting how the carrying capacity is realised. In this formulation, *β*_0_ and *δ*_0_ denote the intrinsic birth rate and intrinsic death rate, respectively, in an initial small population. We assume *β*_0_ > *δ*_0_. The actual birth and death rates are density-dependent as:

$$\;\beta (n_{i,j})=\;\beta_{0}-\;n_{i,\cdot }(\;\beta_{0}-\;\theta )/\;K_{j}$$ (1a)

$$\;\delta (n_{i,j})=\;\delta_{0}+\;n_{i,\cdot }(\;\theta -\;\delta_{0})/\;K_{j},$$ (1b)

where $\;n_{i,\cdot }$ denotes the total population in patch *i*, and a parameter *θ*, such that *β*_0_ ≥ *θ* ≥ *δ*_0_, determines in what proportion the rates are changed with population size.

With *θ* = *β*_0_ the birth rate stays constant and the death rate increases with population density, and thus the cells have a shortened expected lifetime at carrying capacity. This corresponds to the classical formulations through elementary reactions yielding the logistic growth model, where the carrying capacity is realised by increasing mortality due to intraspecific competition between the cells. If we set *θ* = *δ*_0_, the death rate stays constant and the birth rate is reduced. Thus, the expected lifetime of cells stays the same at carrying capacity, while the number of births, and cell population turnover, are reduced. These two modes to realise the carrying capacity can be linked to the ecology of cancer. Increase of death rate at the carrying capacity maps onto so called hazards such as immune cells, toxins and the accumulation of waste products in the tumour micro-environment, whereas reduction of birth rate corresponds to changes in resources such as diminishing of oxygen, glucose, micronutrients and growth signals [28].

For simplicity, we study these two extreme cases. While both effects probably happen, no estimates of their proportion exist to our knowledge. Any *θ* value will yield the same mean field model for population growth, but will affect the number of births, and thus the opportunities for driver mutations.

### Mathematical model of treatments

We study the benefit in targeting either the birth rate or the death rate by medication. To this end, we assume that either rate can be affected, by some amount Δ, and compare the two modes, reducing the intrinsic birth rate (*β*_0_ → *β*_0_ − Δ, cytostatic treatment) and increasing the intrinsic death rate (*δ*_0_ → *δ*_0_ + Δ, cytotoxic treatment), against each other, given that the change in overall growth rate is the same.

We consider two settings in the management of solid tumours and hematological malignancies. First, the treatment reduces cancer cell growth, but the overall rate stays positive so that the therapy can only (substantially) extend the waiting time until progression. Second, therapy can cause the primary tumour to decline, but metastatic and mutated cancer cells still exhibit positive growth.

In the first setting, treatment may change the cell turnover at equilibrium, but not the equilibrium itself, which remains unchanged at the carrying capacity *K*. The effect of the treatment depends on how the carrying capacity is realised (by parameter *θ*). The birth and death rates under each treatment combination are listed in Table 2. This first scenario corresponds to containment strategy [23, 29], where aggressive treatment options are considered undesirable due to various reasons, and instead the objective is to maintain a low cancer cell population size as long as possible. We further note that the implementation of the therapy could also affect the equilibrium carrying capacity itself. For example, this would happen if we assume that the competition terms (i.e. those multiplied by $\frac{\;n_{i,\cdot }}{K_{j}}$) do not change due to treatments (see Table 2). In such a case, the carrying capacity *K* would also respond to therapy resulting in a new lower value $K^{\prime }=\frac{\left(\beta_{0}-\Delta -\delta_{0}\right)}{\beta_{0}-\delta_{0}}K$. This reduction is the same for all cases considered here so that the results we present hold for such an implementation of therapy as well (with *K* replaced by *K*′).

**Table 2:** Treatment effects. The birth and death rates under treatment depend on whether the carrying capacity is implemented via increasing death rate (*θ* = *β*_0_) or decreasing birth rate (*θ* = *δ*_0_).

model	cytostatic (*β*_0_ → *β*_0_ − Δ)	cytotoxic (*δ*_0_ → *δ*_0_ + Δ)
*θ* = *δ*_0_	$\beta \left(n_{i,j}\right)=\left(\beta_{0}-\Delta \right)-\frac{\;n_{i,\cdot }}{K_{j}}\left(\beta_{0}-\Delta -\delta_{0}\right)$	$\beta \left(n_{i,j}\right)=\beta_{0}-\frac{\;n_{i,\cdot }}{K_{j}}\left(\beta_{0}-\Delta -\delta_{0}\right)$
	*δ*(*n*_*i*,*j*_) = *δ*_0_	*δ*(*n*_*i*,*j*_) = *δ*_0_ + Δ
*θ* = *β*_0_	*β*(*n*_*i*,*j*_) = *β*_0_ − Δ	*β*(*n*_*i*,*j*_) = *β*_0_
	$\delta \left(n_{i,j}\right)=\delta_{0}+\frac{\;n_{i,\cdot }}{K_{j}}\left(\beta_{0}-\Delta -\delta_{0}\right)$	$\delta \left(n_{i,j}\right)=\left(\delta_{0}+\Delta \right)+\frac{\;n_{i,\cdot }}{K_{j}}\left(\beta_{0}-\Delta -\delta_{0}\right)$

In the second setting, the resident cancer cell population within the primary tumour declines exponentially towards zero, and carrying capacity does not affect it. Birth and death reactions still take place in the population, and are affected by the chosen treatment, despite negative overall growth. The metastatic and mutated populations express positive growth, and are treated similarly to the first scenario. This second scenario considers resistance to treatment where the cancer cells escape medication through driver mutations and metastasising to different tissue [30]. In both settings, we assume that surgical removal is not an option for the management of solid tumours. This is the case for patients who are inoperable, e.g. due to other health conditions, or whose primary tumours are unresectable as is often the case in advanced tumours of the brain, lung, liver or pancreas. Furthermore, we note that technology for detection is constantly improving so that interventions will happen earlier in the future. As a consequence, what is a typical first line of therapy might change from invasive surgery to chemotherapy or immunotherapy.

### Analytical waiting times for tumour progression: Case no decline

We first derive approximate waiting times until the last stage of tumour progression for the cell population model defined above. If effective driver mutation and migration rates are much smaller than intrinsic birth and death rates, i.e., *μ K*, *γ K* ⪡ *β*_0_ − *δ*_0_ − Δ, we can assume time-scale separation: patch cell populations reach their carrying capacities much faster than driver mutations and migration events are generated. In this case, the first successful mutation or migration event, whichever takes place first, leads to treatment failure. Furthermore, in our mathematical analysis, we neglect the competition between driver mutation and the primary tumour cell population (we note that competition is taken into account in our simulations). In this setting, we can measure the efficacy of treatment as the change in expected time until the first successful mutation or migration.

While at carrying capacity, cells in the primary tumour migrate at rate *μ K* and acquire driver mutations at rate *γ β* (*K*) *K* = *γ θ K*. Thus, the expected time until the first event, either migration or mutation, is 1/(*K*(*γθ* + *μ*)). The extinction probability of new population is *δ*_0_/*β*_0_ [10], and the mean number of mutations and migrations until one of them escapes extinction is *β*_0_/(*β*_0_ − *δ*_0_) (given failure probability *p*, the mean number of trials until the first success is 1/(1 − *p*)). The expected time until first successful migration or mutation is thus given by

$$\left\langle T\right\rangle =\frac{1}{\;K(\;\gamma \;\theta +\;\mu )}\cdot \frac{\;\beta_{0}}{\;\beta_{0}-\;\delta_{0}}.$$ (2)

While both cytostatic and cytotoxic treatments lead to identical reduction in net growth rate, and do not change *K*, their impact on the tumour dynamics differs depending on driver mutation, metastasis, intrinsic birth and death rates, and the details of cell competition at the carrying capacity.

These dependencies can be analysed by comparison of the expected waiting times of progression. For the scenario where intra population cell competition is due to death rate increasing at the carrying capacity, cytostatic treatment gives

$$\left\langle T_{\;\theta \to \;\beta_{0}-\;\Delta ,\;\beta_{0}\to \;\beta_{0}-\;\Delta }\right\rangle =\frac{1}{\;K(\;\gamma (\;\beta_{0}-\;\Delta )+\;\mu )}\cdot \frac{\;\beta_{0}-\;\Delta }{\;\beta_{0}-\;\delta_{0}-\;\Delta }$$ (3)

and cytotoxic gives

$$\left\langle T_{\;\theta \to \;\beta_{0},\;\delta_{0}\to \;\delta_{0}+\;\Delta }\right\rangle =\frac{1}{\;K(\;\gamma \;\beta_{0}+\;\mu )}\cdot \frac{\;\beta_{0}}{\;\beta_{0}-\;\delta_{0}-\;\Delta }.$$ (4)

Similarly, for the scenario where intra population cell competition is due to birth rate decreasing at the carrying capacity, cytostatic treatment gives

$$\left\langle T_{\;\theta \to \;\delta_{0},\;\beta_{0}\to \;\beta_{0}-\;\Delta }\right\rangle =\frac{1}{\;K(\;\gamma \;\delta_{0}+\;\mu )}\cdot \frac{\;\beta_{0}-\;\Delta }{\;\beta_{0}-\;\delta_{0}-\;\Delta }$$ (5)

and cytotoxic gives

$$\left\langle T_{\;\theta \to \;\delta_{0}+\;\Delta ,\;\delta_{0}\to \;\delta_{0}+\;\Delta }\right\rangle =\frac{1}{\;K(\;\gamma (\;\delta_{0}+\;\Delta )+\;\mu )}\cdot \frac{\;\beta_{0}}{\;\beta_{0}-\;\delta_{0}-\;\Delta }.$$ (6)

The ratios of waiting times until progression showing optimal treatment scenarios under each parameter combination, are derived in Materials and Methods (Eqs 12 and 13). We note that the analytical waiting time ratios depend on the parameters *μ* and *γ* only via *μ*/*γ*, and thus it suffices to estimate their relative magnitudes to optimise therapy.

**Fig 2:**
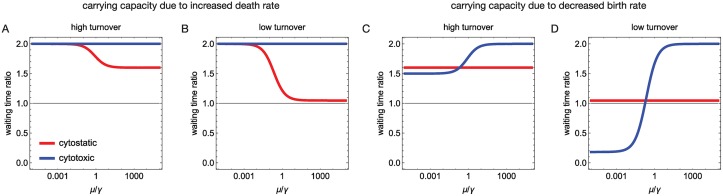
Case primary tumour does not decline. Ratios of expected waiting times until progression of cytostatic (red, target *β*_0_) and cytotoxic (blue, target *δ*_0_) compared to no therapy, under different migration *μ* and driver mutation rates *γ* from Eqs 2–6; black this line denotes equally long waiting times (i.e the ratio = 1). High turnover cases have (*β*_0_ = 1.0, *δ*_0_ = 0.6) and low turnover cases (*β*_0_ = 0.42, *δ*_0_ = 0.02), treatment strength Δ = 0.2. **A,B** High and low turnover cases where cell competition at the primary tumour is due to increasing death rates. Both treatments are better than no treatment, and cytotoxic (blue) is superior to cytostatic (red) treatment. **C** High turnover case where cell competition at the primary tumour is due to decreasing birth rates. Both treatments are better than no treatment and cytostatic treatment is better than cytotoxic when *μ*/*γ* < (*β*_0_ − *δ*_0_ − Δ). **D** Low turnover case where cell competition at the primary tumour is due to decreasing birth rates. Cytotoxic treatment is better than cytostatic treatment when *μ*/*γ* > (*β*_0_ − *δ*_0_ − Δ) and better than no treatment if *μ*/*γ* > (*β*_0_ − 2 *δ*_0_ − Δ). Cytostatic treatment is always better than no treatment. For each case, at least one of the treatments is better than no therapy.

To build intuition in what follows, we further note that Eqs 3–6 each consist of two parts, a term related to generation of events of mutation and migration and a term related to their probability of escaping stochastic extinction. For all cases where metastasis dominates driver mutation, i.e. *μ*/*γ* ≫ 1, the first term is approximately equal whereas the second term responds better to the cytotoxic therapy. This gives an increase of waiting time until progression by a factor $\frac{\beta_{0}}{\beta_{0}-\Delta }$ for cytotoxic therapy over the cytostatic therapy (Fig 2). In other words, cytotoxic therapy is always better in suppressing the establishment (i.e. escaping stochastic extinction) of events compared to a cytostatic therapy. However, when mutations start to play an increasingly important role, i.e. *μ*/*γ* ≲ 1, this increased suppression effect can become less important than how the therapies affect the number of births (Fig 2). We now consider in detail this trade-off between influencing births at the carrying capacity versus increasing stochastic extinction for various parameter regimes leading to different optimal treatments.

#### Treatments versus no treatment

Cytostatic treatment is always better, as it increases the waiting time to cancer progression, compared to no treatment (Eqs 3 and 5, Fig 2). The improvement is due to the therapy both reducing the establishment probability of events and reducing (or keeping constant) the number of births at the carrying capacity when compared to no therapy. The improvement is independent of *μ* and *γ* when the carrying capacity is realised by decreasing birth rate, because then cytostatic therapy does not change the number of births at *K*, and vanishes for low turnover tumours (*δ*_0_ → 0). If carrying capacity is due to increasing death rate, the improvement depends on the ratio *μ*/*γ* as then cytostatic therapy changes the number of births at *K*.

For cytotoxic treatment the picture is more delicate (Fig 2). Similarly to cytostatic therapy, cytotoxic therapy reduces the establishment probability of events compared to no therapy but the impact in births is varied. If carrying capacity is realised via increasing death rate, cytotoxic treatment is always better compared to no treatment and the efficacy does not depend on *μ* and *γ*, because then cytotoxic therapy does not change the number of births at *K*. However, when the carrying capacity is realised by decreasing birth rate, two qualitatively different regimes emerge. In high turnover tumours (i.e. *δ*_0_ ≥ (*β*_0_ − Δ)/2) cytotoxic treatment is again always better compared to no treatment, but for low turnover tumours (*δ*_0_ < (*β*_0_ − Δ)/2) the effect can reverse. In these cases cytotoxic treatment harms the patient when *μ*/*γ* < (*β*_0_ − 2 *δ*_0_ − Δ) (Fig 2D). This effect is due to cytotoxic treatment inadvertently increasing the evolutionary potential of the tumour by creating more space for births to occur and thus expediting driver mutation generation.

#### Cytostatic versus cytotoxic treatment

If carrying capacity is realised via increasing death rate, cytotoxic treatment is always better or equal than cytostatic treatment (Fig 2A and 2B). This means that the effect of cytotoxic therapy reducing the establishment probability of events can only be matched but not overcome by cytostatic therapy reducing births at the carrying capacity.

For tumours where cell competition leads to decreasing birth rate at the carrying capacity, cytotoxic treatment is better than cytostatic as long as *μ*/*γ* > (*β*_0_ − *δ*_0_ − Δ) (Fig 2C and 2D). We note that this condition happens before cytotoxic treatment becomes worse than no treatment, thus for each case, at least one of the treatments is better than no therapy. As discussed earlier, this subtle behaviour stems from the cytotoxic treatment inadvertently increasing the evolutionary potential of the tumour by creating more space for births to occur.

### Analytical waiting times for tumour progression: Case primary tumour declines

Above, we assumed that while the therapy could reduce the overall growth rate of the primary tumour, it was not able to force it to regress. As shown in Fig 2, such therapies can increase the waiting time to tumour progression substantially, even if they do not allow for eradication in practice. (There is a vanishingly small probability for a large primary tumour to go extinct via fluctuations when intrinsic growth rate is positive [31]). We now consider how efficacy and choice of therapy are affected if the primary tumour can be effectively targeted such that it starts to regress. For a shrinking tumour of size *n*, the concept of a carrying capacity is not biologically sensible, and we assume that its cell population decays exponentially with a rate *r*_0_ < 0 while emitting escape events with rate $\Theta \left(t\right)=\left(\beta_{\Delta }^{\text{primary}}\;\gamma +\;\mu \right)(1-\delta_{\Delta }/\beta_{\Delta })n\left(t\right)$. Where the first term contains driver mutation rate and migration rate of the primary, the second the probability to escape stochastic extinction, and *n*(*t*) denotes the time dependent population size of the declining primary tumour. The birth and death rates will take their under therapy values depending on the applied therapy, denoted by subindex Δ. As here the primary tumour will respond to the therapy differently than the driver or metastasis events (so that it can decline), we further define $\beta_{\Delta }^{\text{primary}}$ to denote the birth rate at the primary tumour under the applied therapy.

Clearly, if |*r*_0_| is large compared to the total rate of escape via metastasis or driver mutation generation, the tumour essentially always becomes eradicated, and the therapy choice is between two almost equally good options. Thus we are mostly interested in the case where *r*_0_ is negative but small in magnitude. We note that the compound process of successful escape events is now an inhomogeneous Poisson process in time as the primary tumour has its own (approximately deterministic) dynamics. The process can be analysed by transforming time by *t*′(*t*) = log(1 + *r*_0_*t*)/*r*_0_, which makes it again homogenous, and evaluating the expected time to first successful event conditioned that one happened

$$\left\langle T\right\rangle =Z^{-1}\int_{t_{0}}^{t_{\text{max}}}t^{\prime }\left(s\right)\Theta \left(t_{0}\right)\text{exp}\left(-\Theta \left(t_{0}\right)s\right)\;\text{d}s$$ (7)

where *Z* = 1 − exp(Θ(*t*_0_)/*r*_0_) is the probability that an escape event did happen before time *t*_max_ = −1/*r*_0_. Integration boundary *t*_max_ denotes the point after which the waiting time for the next event diverges and would cause the unconditional expectation value of the waiting time to diverge as well. The probability that the therapy is successful in eradicating the tumour is then

$$P=\text{exp}(\frac{\Theta (t_{0})}{r_{0}})=\text{exp}\frac{\left(\beta_{\Delta }^{\text{primary}}\;\gamma +\;\mu \right)(1-\delta_{\Delta }/\beta_{\Delta })K}{r_{0}},$$ (8)

so that when |*r*_0_| ≳ Θ(*t*_0_) the therapies start to be very effective.

**Fig 3:**
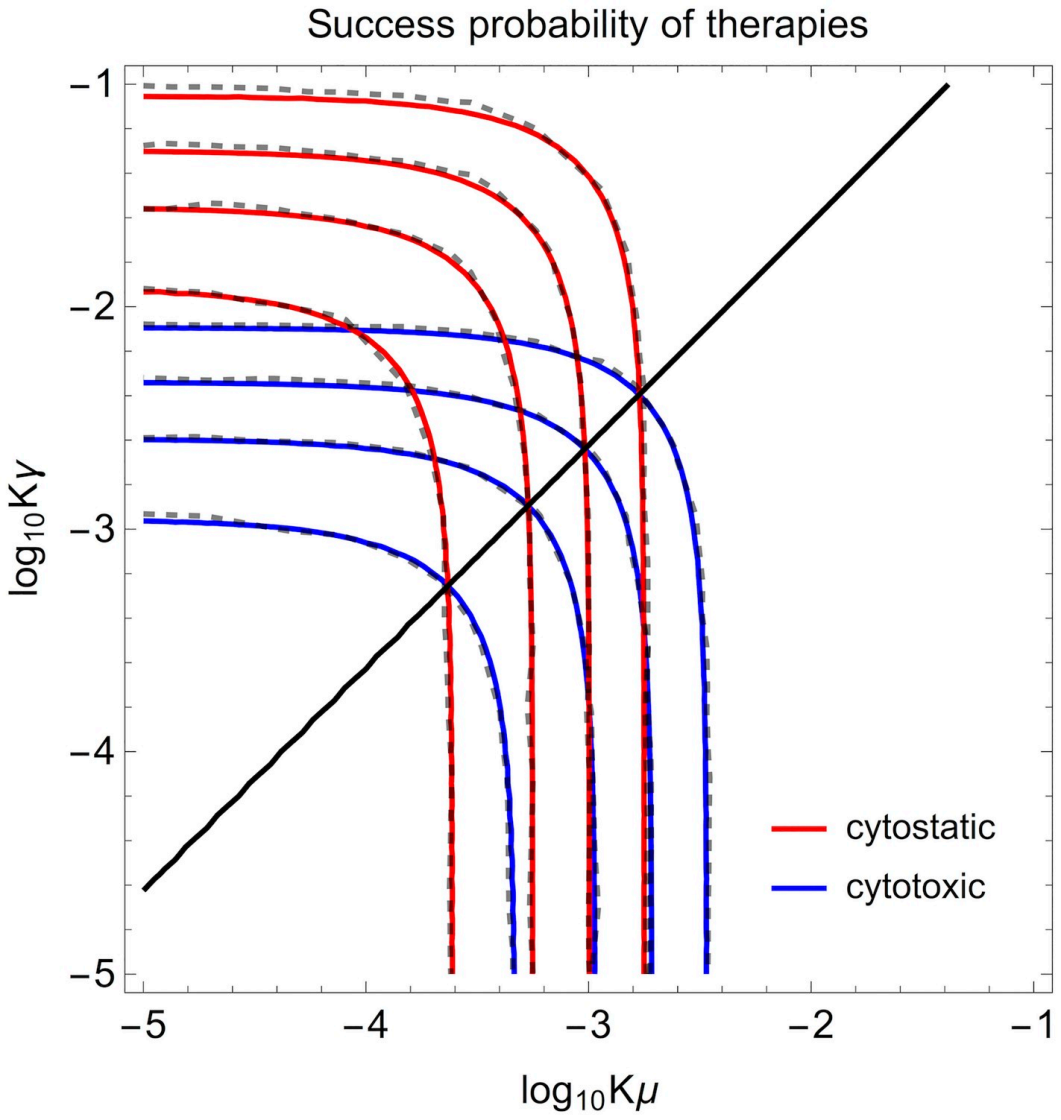
Case primary tumour declines. Isocontours for therapy success (0.2, 0.4, 0.6, 0.8, with upmost contours representing smallest values) from Eq 8 for cytotoxic treatments (blue) and cytostatic treatments (red). Shown is the plane in metastasis rate (*K μ*) and mutation probability (*K γ*) multiplied by the population size at carrying capacity. Black thick line shows the transition from cytostatic better (left side) to cytotoxic better (right side) which takes place at *β*_0_*γ* = *μ*. Low turnover case (*β*_0_ = 0.42, *δ*_0_ = 0.02, Δ = 0.2001), simulation data plotted with dashed lines. Primary tumour decayed with rate *r*_0_ = −0.001.

Comparing the ratios of success probabilities under cytotoxic and cytostatic treatments reveals that cytotoxic is better than cytostatic when *β*_0_*γ* < *μ* (Fig 3), where to keep the relation compact we have assumed the primary tumour to respond to cytostatic therapy by $\beta_{\Delta }^{\text{primary}}\to \;\beta_{0}-2\Delta$. Similarly to the containment case, this boundary reflects the trade-off between suppression of establishment of events where cytotoxic is better versus suppression of new mutations where cytostatic is better. The difference between the options is biggest for small |*r*_0_| and vanishes when *r*_0_ ≪ 0 when both treatments are very effective and eradicate the cancer cell population with probabilities approaching one.

### Waiting times for tumour progression via simulation

We tested our analytical results via extensive numerical simulations. The two cases with and without primary tumour declining were simulated via a hybrid stochastic approach (see Materials and methods for details). The results confirm our mathematical analysis (Fig 3 and Supplementary S1 Fig). In addition, we simulated the seeding of a metastasis with a cluster of 50 cells as some cancer metastases can be actually initiated by clusters of 2–50 cells [32]. Seeding a metastasis with a number of cells at a level where the stochastic extinction risk is all but vanished led to loss of efficacy of either treatment against metastatic progression.

## Discussion

We contrasted the impact of two most fundamental treatments, cytostatic and cytotoxic, on tumour progression, while keeping their net effect on tumour growth constant. Based on our mathematical analysis, choosing a specific mode of action lead to a substantial efficacy gain (Fig 2) under particular cell population dynamics. The reason for this gain is in the trade-off between how therapies affect suppression of the establishment of events versus how the therapies affect the number of births.

If the treatment causes the primary tumour to decline, cytotoxic therapy is better than cytostatic when the rate of generating migrations substantially exceeds that of driver mutations. The precise boundary to select the optimal treatment depends on how strongly the primary tumour responds to the therapies (e.g. in Fig 3, where the primary is assumed to be affected by 2Δ, the boundary is at *β*_0_*γ* = *μ*). The relative advantage disappears when the primary tumour declines quickly, and both strategies result in effective cancer eradication.

More interesting differences between the strategies arise in the case of moderate growth reduction, where the tumour cannot be eradicated, either because of lack of efficacy or toxicity reasons, but where the progression can be substantially delayed. This scenario falls under the emerging paradigm of tumour containment, or adaptive therapy [22]. The importance of adaptive therapy could increase with the improvements in early detection of cancers so that invasive intervention by surgical removal may not be preferable as the first line of therapy. Again, based on our model, cytotoxic therapy is superior when metastasis is the predominant mode for progression as measured by waiting times. However, depending on the mode of cell competition and intensity of cell turnover, cytotoxic therapy can even become harmful, and cytostatic therapy should be chosen instead. The harmful effect of increasing the number of cell deaths arises under a low turnover cell population where competition also limits cell reproduction. In this scenario, a cytotoxic agent creates space for new cells to be born, and thus increases the risk of new driver mutations. Similarly, it has been suggested that apoptosis of cancer cells generates space for more aggressive sub-clones, and therefore promotes tumour evolution [33].

While many of the mathematically derived control strategies may be difficult to utilise in clinical practice due to complexity such as time dependent dosaging, our results show clear differences that could be readily implemented. However, application would still need characterisation of the underlying cell population dynamics: birth and death rates, relative strength of driver mutation and metastasis rates, and details of cell competition. Some rough estimates are available from literature. Božić *et al*. [34] have suggested a driver mutation probability of ∼ 3.4 ⋅ 10^−5^ per cell division, based on the ∼34k positions in the human genome that could putatively give rise to a driver mutation, and base point mutation rate of ∼ 5 ⋅ 10^−10^ for colorectal cancer [35]. However, for a big event, a hallmarks of cancer type of driver mutation, the probability per cell division is most likely several orders of magnitude smaller. The rate of metastasis seeding is harder to estimate, since seeded but stochastically extinct metastases are not clinically detectable. Jones *et al*. [35] estimate an average of 1.8 years between an advanced carcinoma founder cell and a succeeding liver metastasis founder cell.

Intrinsic cell birth and death rates in cancer have not been systematically measured thus far. Jones *et al*. [35] estimated a division rate of approximately 0.25 *d*^−1^, in the absence of cell deaths. A wide range of estimates for the ratio of death to birth rates have been presented, ranging from *q* = 0.99 in early tumours [36] and *q* = 0.72 in fast growing colorectal cancer metastases [34] to *q* between 0.1 and 0.5 in chronic myeloid leukaemia [15]. Together, these estimates yield death rates between 0.25 *d*^−1^ to 0.025 *d*^−1^. There are indications that tumours with a high overall growth rate also experience more cell deaths and a higher turnover [37]. However, this wide range underlines the need for modelling the dynamics at the individual level and accounting for stochastic effects as done here. This aspect is often overlooked by analyses focusing on compound growth rates. Finally, presently we can only guess how competition affects these individual rates as resources become limiting at different stages of tumour progression. Without more data on rates of birth and death or modes of competition, we can only say that cytotoxic therapy is superior when metastasis is the predominant mode for progression.

For simplicity, we have analysed only the pure modes of drug action. While drugs with a predominant mode of action do exist, many actual drugs display both cytotoxic and cytostatic qualities in different proportions [9], depending on factors such as dosage and the cell cycle phase at the time of administration. Mainly cytotoxic drugs often cause cell death through apoptosis, autophagy, or regulated necrosis [38], and include mitotic poisons, DNA-reactive drugs, inhibitors of DNA replication, and modulators of DNA topology as major groups [8]. Platinum-based therapies such as cisplatin and less potent but better tolerated carboplatin are examples of cytotoxic drugs, which function by triggering apoptosis [39]. The use of targeted cytostatic drugs started in the late 1990s for treatment of breast cancer [40], against which cytostatics are still commonly used [41]. Currently, cytostatics are rarer than cytotoxic drugs: a recent high-throughput study found 2327 cytostatic-only compounds in a total of 388,000 compounds screened [42]. Cytostasis is often achieved by interference with the signal transduction process, with the largest group of drugs targeting receptor tyrosine kinases [8]. Rapamycin and its analogs are examples of mainly cytostatic drugs [43]. In experiments, rapamycin analogs induced tumour regression in renal cancers, yet the tumours started to regrow when the treatment was discontinued [44]. Dosage and other factors determine the actual magnitudes of cytotoxic and cytostatic effects of these drugs (modelled here by parameter Δ, which to contrast the therapies, was chosen to be equal for both modes of action).

Our results are based on a minimal mathematical model of an evolving cell population and thus can provide insight beyond cancer. A number of studies emphasize the importance of accounting for the relationship between cell turnover components and drug type for the clearance of bacterial infections. Coates et al., among others, have suggested that as bacterial extinction probability is determined by the ratio of the death and growth rates, it can be maximized by combining bactericidal antibiotics (increasing death rate) with bacteriostatic antibiotics (decreasing birth rate) [45]. However, bacteriostatic antibiotics (inducing stasis) can cause tolerance against bactericidal antibiotics (targeting rapidly growing cells) [46], and such antagonism between bactericidal and bacteriostatic antibiotics has been reported to be common [47]. This suggests that combining the two is not a universal solution, and thereby we need to understand the conditions determining the efficacy of each drug type.

Our results highlight the need to carefully measure key evolutionary parameters across cancer types and progression stages in order to utilise therapies effectively. In practice, these parameters are still largely unknown. As a concrete first step, in addition to net growth, it would be helpful to track cell deaths, which could be done *in vitro* cell models via a suitable marker, and was recently achieved *in vivo* by using stable isotopic labeling with deuterated water to measure directly the effects of *ibrutinib* on leukemia cell proliferation and death in 30 patients with CLL [48]. How to measure modes of cell competition cannot be resolved in an *in vitro* assay. As a first step we would need to collect time series data of tumour growth from early on while tracking cell death. This could be pursued first in xenograft or 3D tissue models. For the final parameters of driver mutation and metastasis rates a way forward would be devising epidemiological studies that keep track of primary vs. metastasis tumour burden. Also high resolution monitoring of metastasis formation versus primary tumour growth in a model system could help. Clearly, to properly quantify these cancer biology parameters requires a substantial amount of work. Fortunately, vast amounts of data are already being generated by high-throughput technologies such as sequencing, massively parallel phenotyping assays, lineage tracing, and high-content imaging. In the future, these and other data streams should be combined to measure the missing parameters.

## Materials and methods

### Simulation model

Our simulation model is essentially a patch model where a) the number of patches and b) the number of different populations within a patch can increase through metastases and mutations, respectively. Each migration event initiates a new patch and patches are considered fully independent of each other once initiated. In a migration event, a single cell is transferred to the new patch. Each mutation initiates a new population (i.e. phenotype), and populations within the same patch face competition and density dependent growth. For simplicity, we consider all mutated populations similar, with carrying capacity *L* higher than the resident populations’ carrying capacity *K*.

Populations smaller than a threshold value of 100 cells are simulated via a stochastic simulation algorithm (SSA) following the reactions listed in Table 1. These undergo individual birth and death reactions (and may give rise to new metastases and migrations), and are thus susceptible to stochastic extinction, where the population size hits the zero absorbing boundary. Populations as well as patches may go extinct. The propensities for discrete reactions are calculated as follows:

$$\begin{array}{cc} \text{birth}\; & \left\{ \begin{array}{cc} (1-\;\gamma )(\;\beta_{0}-\frac{\;\beta_{0}-\;\theta }{\;K}\sum_{k}n_{i,k})n_{i,j} & \text{if}\;\;\beta_{0}-\;\Delta -\;\delta_{0}>0\\ (1-\;\gamma )\beta_{\Delta }^{\text{primary}}n_{i,j} & \text{if}\;\;\beta_{0}-\;\Delta -\;\delta_{0}\leq 0 \end{array}\right. \end{array}$$ (9a)

$$\begin{array}{cc} \text{death}\; & \left\{ \begin{array}{cc} (\;\delta_{0}+\frac{\;\theta -\;\delta_{0}}{\;K}\sum_{k}n_{i,k})n_{i,j} & \text{if}\;\;\beta_{0}-\;\Delta -\;\delta_{0}>0\\ \delta_{\Delta }^{\text{primary}}n_{i,j} & \text{if}\;\;\beta_{0}-\;\Delta -\;\delta_{0}\leq 0 \end{array}\right. \end{array}$$ (9b)

$$\text{migration}\;\;\mu n_{i,j}$$ (9c)

$$\begin{array}{cc} \text{mutation}\; & \left\{ \begin{array}{cc} \;\gamma (\;\beta_{0}-\frac{\;\beta_{0}-\;\theta }{\;K}\sum_{k}n_{i,k})n_{i,j} & \text{if}\;\;\beta_{0}-\;\Delta -\;\delta_{0}>0\\ \;\gamma \beta_{\Delta }^{\text{primary}}n_{i,j} & \text{if}\;\;\beta_{0}-\;\Delta -\;\delta_{0}\leq 0\;, \end{array}\right. \end{array}$$ (9d)

where the cases with *β*_0_ − Δ − *δ*_0_ ≤ 0 apply for the primary in *primary tumour declines*-scenario.

All populations *i*, *j* above the threshold value were simulated by using a system of ODEs:

$$\frac{\;\text{d}}{\;\text{d}t}n_{i,j}=(\;\beta_{0}-\;\Delta -\;\delta_{0})n_{i,j}-\frac{\;\beta_{0}-\;\Delta -\;\delta_{0}}{\;K_{j}}n_{i,j}\sum_{k}n_{i,k},$$ (10)

where the competition term is removed for primary tumour in *primary tumour declines*-scenario.

Euler’s method of the second degree was used for numerical treatment of Eq 10. Note that migration and mutation events generated by large populations were still treated as discrete reactions, handled via the SSA.

The hybrid simulation scheme operates as follows. First, all reaction propensities of all populations are calculated according to Eq 9, and their sum is assigned to total propensity λ_tot_(*t*). Then, the system of ODEs given by Eq 10 is advanced, while simultaneously updating λ_tot_(*t*), until the following condition is reached:

$$\int_{t}^{t+\tau }\;\lambda_{\text{tot}}\;(s)\;\text{d}s=y,$$ (11)

where *y* ∼ Exp(1). With time dependent propensities we do not know beforehand the (exponentially distributed) waiting time until the next reaction, but to the same effect we can integrate the propensity sum until we reach a value picked from the unit exponential distribution [49]. At this point, a single discrete reaction is sampled based on propensities, and resolved. This scheme is repeated until a stop condition (total population extinction, treatment failure through n_crit_, maximum simulation time) is reached. n_crit_ defines the total cancer cell population size at which we consider a simulation lost through treatment failure. The value used in simulations was n_crit_ = 1.5 *K*.

The simulation scheme is the same as in [49] except that partitioning to discrete and continuous reactions is handled differently. We consider migration and mutation events always discrete, and birth and death reactions discrete whenever the population size is under a set threshold. Treating large populations as continuous greatly speeds up the simulations, but disregards population fluctuations. These large population fluctuations are of minor importance in our case, since a) probability of stochastic extinction of a population above threshold is exceedingly small, and b) waiting time until a relatively rare event (small *μ*, *γβ*) is dependent on average population size over time.

### Code availability

The simulation codes are available from github.com: https://github.com/mustonen-group/contrasting-cytotoxic-cytostatic.

### Parameters

Simulations were carried out with two turnover values: high (*β*_0_ = 1.0, *δ*_0_ = 0.6) and low (*β*_0_ = 0.42, *δ*_0_ = 0.02) (Fig 1). Both cases have the same growth rate (*r* = *β*_0_ − *δ*_0_ = 0.4), but different stochastic extinction risk (*q*_H_ = 0.6, *q*_L_ = 0.047). The carrying capacity was set to *K* = 10^9^ (except for fully stochastic simulations, see below), however, this models also smaller tumours equally well as only the products *Kμ* and *Kγ* are of relevance for both the mathematical analysis and simulation. This is because we do not take into account stochastic fluctuations of the populations at the carrying capacity. These can be neglected as the probability of stochastic extinction of a population at *K* is very small (with our parameters for any reasonable tumour size > 10^2^) and the waiting time until a relatively rare epoch defining escape event depends on the average population size over time. The threshold population size above which populations were treated as continuous was set to 100. The new (fatal) carrying capacity *L* was set to 10*K* but could also be 100*K* or even more without it changing the results. This is because the stopping condition at n_crit_ = 1.5 *K* ensures that very little or no growth suppression is felt by the escape events due to carrying capacity *L*. Note, we could equally well require the tumours to grow all the way up to *L* and the results would stay essentially the same with a small extra time representing that growth added to the waiting times of both processes.

In the containment strategy case, i.e. treatment insufficient to eradicate the tumour, the effect of treatment was set to Δ = 0.2, effectively halving the growth rate for all patches. In the case where the primary tumour could be made to decline, the treatment’s effect was Δ = 0.401 on the primary tumour, yielding an effective growth rate of *r* = −0.001. Here, there effect of medication to metastatic patches and mutated populations was still Δ = 0.2, providing positive growth and a possible escape route for cancer.

Additionally, the system was simulated using the full stochastic model without any continuously treated populations. Here, a smaller carrying capacity *K* = 1000 was used. The median waiting times obtained by this method very similar to the values given by the hybrid stochastic simulation, thus validating the simulation scheme.

### Comparisons of analytical waiting times until progression

From Eqs 2–6 we can derive the ratios of waiting times to compare treatment options under the two different carrying capacity model assumptions. Under the model *θ* = *β*_0_, i.e. death rate increases towards carrying capacity, we have:

$$\frac{\left\langle T_{\text{cytostatic}}\right\rangle }{\left\langle T_{\text{no}\;\text{treatment}}\right\rangle }>1\;\text{when}\;\;\mu \;\delta_{0}>\;\gamma \;\beta_{0}(\;\Delta -\;\beta_{0})$$ (12a)

$$\frac{\left\langle T_{\text{cytotoxic}}\right\rangle }{\left\langle T_{\text{no}\;\text{treatment}}\right\rangle }>1\;\text{when}\;\;\Delta >0$$ (12b)

$$\frac{\left\langle T_{\text{cytotoxic}}\right\rangle }{\left\langle T_{\text{cytostatic}}\right\rangle }>1\;\text{when}\;\;\Delta \;\mu >0,$$ (12c)

from which we can see that both treatment options are always better than no treatment, and cytotoxic treatment is always better than cytostatic treatment, given strictly positive parameter values, and *β*_0_ > Δ.

For model assumption *θ* = *δ*_0_, i.e. birth rate decreases towards carrying capacity, we have:

$$\frac{\left\langle T_{\text{cytostatic}}\right\rangle }{\left\langle T_{\text{no}\;\text{treatment}}\right\rangle }>1\;\text{when}\;\;\delta_{0}\;\Delta >0$$ (13a)

$$\frac{\left\langle T_{\text{cytotoxic}}\right\rangle }{\left\langle T_{\text{no}\;\text{treatment}}\right\rangle }>1\;\text{when}\;\;\gamma (\;\beta_{0}-\;\Delta -2\;\delta_{0})<\;\mu$$ (13b)

$$\frac{\left\langle T_{\text{cytostatic}}\right\rangle }{\left\langle T_{\text{cytotoxic}}\right\rangle }>1\;\text{when}\;\;\gamma (\;\beta_{0}-\Delta -\;\delta_{0})>\;\mu$$ (13c)

Here, we see that cytostatic treatment is always better than no treatment, and while cytotoxic treatment can be more effective than cytostatic, especially under high metastasis rate *μ* and low mutation probability *γ*, cytostatic treatment can be worse than no treatment at all if mutation probability is higher and the metastasis risk is low.

## Supporting information

S1 Fig Waiting times for tumour progression: Case no decline. Isocontours for waiting times for tumour progression (2^1^, …, 2^16^, upmost contours representing smaller values) from Eqs 2–6 with two turnover values, high (*β*_0_ = 1.0, *δ*_0_ = 0.6) and low (*β*_0_ = 0.42, *δ*_0_ = 0.02), and therapy magnitude Δ = 0.2. **A** no treatment **B** cytostatic treatment and **C** cytotoxic treatment. Simulation results, 10^4^ independent runs per *μ*, *γ* pair, are shown in dashed lines. Analytical and simulation results closely agree until about *Kμ*, *Kγ* ∼ 0.01 − 0.1 when the time-scale separation argument starts to break down. We note that the longest waiting time isocontours are underestimated by the simulations due to our stopping condition at 10^5^ steps which truncates an increasing number of the 10^4^ independent runs at very small values of *Kμ* + *Kγ* < 10^−4^.
(EPS)
